# Early experience with transcatheter ventricular septal defects closure with the KONAR-MF multifunctional occluder

**DOI:** 10.3389/fped.2025.1528490

**Published:** 2025-04-07

**Authors:** Abdelrahman Elafifi, Susy Kotit, Mahmoud Shehata, Omar Deyaa, Asmaa Ramadan, Mohammad Tawfik

**Affiliations:** ^1^ Pediatric Cardiology, Aswan Heart Centre, Aswan, Egypt; ^2^ Cardiology, Tanta University, Tanta, Egypt; ^3^ Pediatric Department, Suez Canal University, Ismailia, Egypt

**Keywords:** VSD, percutaneous VSD closure, Konar MF, ventricular septal defect, perimembranous VSD, muscular VSD

## Abstract

**Introduction:**

Transcatheter device closure of ventricular septal defects (VSDs) offers an appealing and effective alternative to surgical repair. The Lifetech™ Konar-Multifunctional Occluder (MFO) VSD occluder has gained increasing application due to its versatility and promising outcomes.

**Objectives:**

We aim to evaluate our experience with the MFO device for VSD closure.

**Methods:**

We conducted a prospective analysis of clinical data from 151 patients who underwent percutaneous closure of muscular and perimembranous VSDs using the MFO device at our institution between November 2018 and September 2023. Comprehensive assessments of safety and procedural outcomes were performed.

**Results:**

The patient's mean age was 55.4 ± 51.6 months (range, 6 months to 31 years), and the mean weight was 17.6 ± 11.9 kg (range, 5–86). Among the patients, 94 (62.3%) had perimembranous defects, while the remaining had muscular VSDs. The mean defect diameter was 4.8 ± 1.5 mm (range, 2–10). The retrograde approach was applied in 133 patients (88.7%). Device implantation was successful in 98.7% of patients. One procedure (0.7%) failed due to device migration, requiring surgical retrieval and VSD closure, and another patient with a significant residual shunt needed placement of an additional device in another session. The mean procedure time was 44 ± 2 min, with a mean fluoroscopy time of 12.8 ± 7.7 min. The mean follow-up duration was 11 ± 9.7 months (range, 6–35). Non-significant shunts were found in 32 patients (21.2%). Newly acquired valve regurgitation was observed in 16 patients (10.6%), including 11 patients (7.3%) with trivial-to-mild aortic regurgitation and 5 (3.3%) with moderate-to-severe tricuspid regurgitation. Electrophysiological adverse events occurred in 5 patients (3.3%), including nodal rhythm (*n* = 3, 2%), intermittent heart block (*n* = 1, 0.7%), and severe bradycardia (*n* = 1, 0.7%). Vascular complications were documented in 13 patients (8.6%) including one developing chronic vascular occlusion.

**Conclusion:**

Percutaneous VSD closure with the MFO device is a safe, effective, and feasible procedure via both antegrade and retrograde approaches.

## Introduction

Transcatheter closure of ventricular septal defects (VSDs) has emerged as an appealing alternative to surgery (1), offering a minimally invasive approach with reduced morbidity, shorter hospital stays, and faster recovery times (2–4) while demonstrating a success rate of 96.6% without an increased risk of significant valvular regurgitation or heart block (1).

This technique has gained widespread acceptance, particularly for muscular VSDs, where it has demonstrated high success rates, with low complication rates (4) and an excellent safety profile. While muscular defects are anatomically simpler and located away from vital structures, closure of perimembranous VSDs presents unique challenges due to their proximity to critical cardiac structures, such as the aortic and tricuspid valves and the conduction system (5, 6), rendering it more difficult to close percutaneously.

Common complications include device migration, aortic (7, 8) and tricuspid valve regurgitation (9, 10), as well as vascular complications (3, 11, 12), while complete heart block remains the most malignant (3, 13).

The Amplatzer Membranous VSD Occluder, was withdrawn from the market due to a high incidence of complete heart block, reported in up to 5%–10% of cases (2). This setback prompted interventionists to explore off-label use of other devices, which yielded promising results with reduced complication rates (14, 15).

In recent years, the Lifetech KONAR-MF™ Multifunctional Occluder (MFO) has emerged as a novel device designed to address the limitations of the previous occluder, receiving its CE mark in May 2018 (16). Since its first report of successful VSD closure in pediatric and adult patients (16) it has shown to minimize risks associated with cardiac conduction system damage (16, 17) while achieving high rates of complete closure and low incidences of significant residual shunts (9, 18, 19) and major complications (10, 18, 20–24) in both muscular and permembranous VSDs. This device has proven particularly effective for closing large VSDs in small children weighing less than 10 kg, presenting a viable alternative to surgical intervention even in this vulnerable population (10, 20).

This study outlines our experiences using the Lifetech™ Konar-MF for the percutaneous closure of perimembranous and muscular VSDs over the past five years to analyze the possible benefits of this device.

## Methods

### Study objectives

We aim to evaluate our experience with the MFO device for percutaneous closure of perimembranous and muscular VSDs.

### Study design and population

We prospectively analyzed data from 151 patients who underwent percutaneous closure of muscular and perimembranous VSDs with the MFO device at our institution between November 2018 and September 2023. Patient demographics, procedural details, and early follow-up outcomes were recorded in a secure database, including all adverse events. Each case was approved by a multidisciplinary team, and patients were informed of all treatment options, including surgery. The study complied with local regulations and institutional requirements, with protocol approval from the Aswan Heart Centre Research Ethics Committee (MYFAHC_MFO). Written informed consent was obtained from participants' legal guardians or next of kin.

#### Inclusion criteria

Indications for closure of perimembranous (with or without a membranous septal aneurysm) or muscular VSD using the MFO device included the following: (1) patients weighing >2.5 kg, (2) patients aged >6 months, (3) echocardiographic evidence of significant cardiac shunting, such as a left ventricular end-diastolic diameter (LVEDD) *z*-score >2.0 or a left atrium-to-aortic diameter ratio >1.5, or (4) clinical symptoms of congestive heart failure, or (5) new-onset or progressive aortic valve prolapse, or (6) history of infective endocarditis associated with the VSD.

#### Exclusion criteria

Exclusion criteria included the presence of one or more of the following: (1) perimembranous VSD (PmVSD) with aortic cusp prolapse and moderate or severe aortic valve regurgitation (AVR), (2) PmVSD with deficient subaortic rim (SAR) less than 2 mm (in non-aneurysmal defects), (3) inlet or subpulmonic (infundibular) VSDs, (4) documented irreversible pulmonary vascular obstructive disease with pulmonary vascular resistance greater than 8 UW, (5) active infective endocarditis or other bacterial infections, (6) other cardiac structural defects requiring surgical intervention, and (7) a history of medical conditions that contraindicate the use of anticoagulants or antiplatelet drugs.

### Pre-procedural planning

All patients underwent routine pre-intervention evaluations, including a 12-lead electrocardiogram (ECG), chest x-ray (CXR), and detailed transthoracic echocardiography (TTE). Standard TTE views (subcostal, apical, and parasternal) were used to assess the defect's morphology and hemodynamic impact. The morphological evaluation included the defect's position, diameter on the left ventricular (LV) and right ventricular (RV) sides, VSD length, and rim dimensions. The specific rims assessed were the subaortic rim (distance between the aortic valve annulus and the defect's superior margin) and the tricuspid rim (distance between the tricuspid valve annulus and the defect's inferior margin). Device size selection was primarily based on meticulous TTE measurements, later confirmed by angiography. Intraprocedural transesophageal echocardiography (TEE) was selectively used for cases with complex anatomy or suboptimal echocardiographic windows.

### Study device

The KONAR-MF multifunctional occluder (Lifetech, Shenzhen, China) is a self-expandable low-profile disc device featuring two discs connected by a waist, with a unique truncated cone shape on the left disc that extends into an arm connecting to the right disc. Both discs are of equal size, and include a hub with a screw for device positioning retrogradely (from the LV side) or anterogradely (from the RV side).

**Figure 1:**
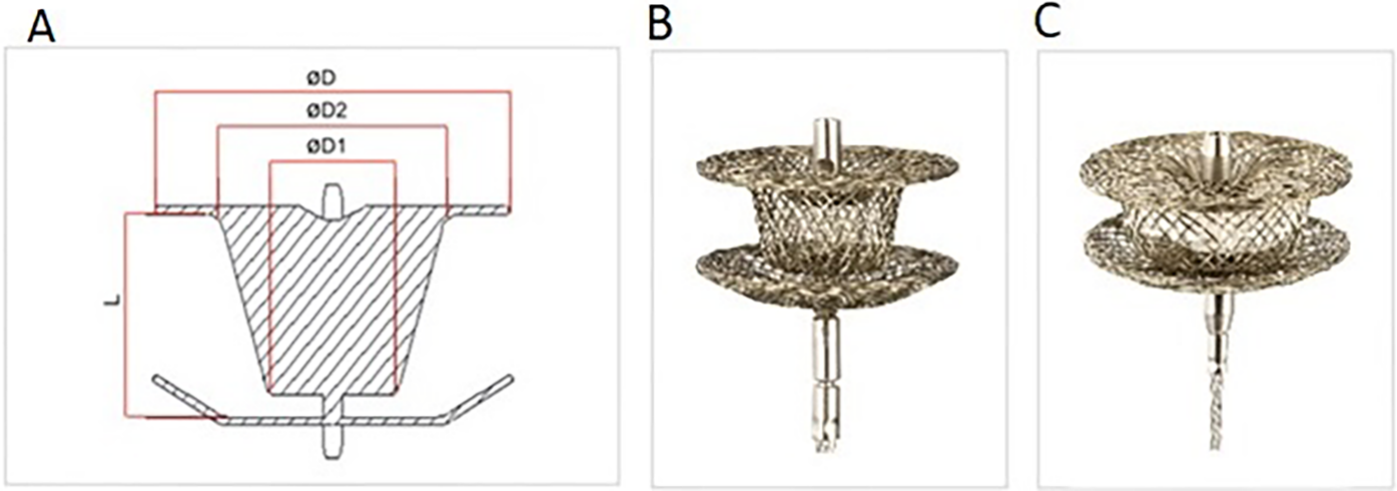
The KONAR-MF multifunctional occluder (Lifetech, Shenzhen, China). **(A)** Device components denomination: ØD = Disc diameter, Ø1 = waist diameter right ventricular side, Ø2 = waist diameter left ventricle side, **(B)** the device **(C)** device with PTFE membrane inside.

The device is available in various sizes, with waist diameters ranging from 5 to 3 mm to 14–12 mm, sewn with polytetrafluoroethylene (PTFE) membranes from size 9–7 mm onwards for improved occlusion (Figure 1). Device delivery employs a SteerEase™ introducer, recommended for a 4–7 Fr sheath or guiding catheter with specific inner lumen dimensions. Bench testing indicates that a sheath one French size smaller than recommended may be sufficient for delivery.

### Device selection

The choice of the appropriate device was guided by the echocardiographic VSD description including VSD type, VSD orifices sizes, the presence of an aneurysm, and the SAR efficiency.

#### Perimembranous ventricular septal defects (pmVSDs)

In perimembranous VSDs with sufficient SAR and absence of deep ventricular aneurysm, device selection was based on a waist diameter of 2 mm greater than the maximum diameter of the right orifice of the defect. In case of a deficient SAR where the left ventricular discs of the device exceed the left ventricular side of the defect, posing a risk of contact with the aorta, device selection was decreased to match the size of the right ventricular disc or to be slightly larger by 1 mm.

For pmVSDs with multiple right orifices, a retrograde approach was preferred for device placement within the aneurysm, to ensure effective occlusion of all right orifices while avoiding interference with the conduction system.

In the presence of a large aneurysm, especially if the SAR is deficient, the selected device was deployed within the aneurysm instead of the LV entrance, with a size that did not exceed the narrowest part of the VSD diameter by 2 mm.

In case of a deep aneurysm (≥7 mm) and/or a right ventricular exit-to-left ventricular entry diameter ratio greater than 0.5, the device was slightly oversized and deployed through an arterio-venous circuit to control the initial LRD deployment within the aneurysm and decrease the risk the AoV encroachment (17).

#### Muscular ventricular septal defects (mVSDs)

For muscular VSDs, devices were positioned over the interventricular septum, with special attention given to high muscular defects to prevent potential complications related to the nearby conduction system. Device selection criteria mirror those for the perimembranous VSDs, with the right ventricular disc size chosen to exceed the defect size by 2 mm.

### Study procedure

The procedure was performed under general or local anesthesia with TTE guidance and fluoroscopic supervision. Access was gained via the femoral artery in all cases, with additional venous access in some. Patients received a single IV dose of heparin (100 IU/kg) and prophylactic IV cefazolin (30 mg/kg) at the start, followed by two additional doses. VSD anatomy was visualized using a pigtail catheter through left ventriculography in long axial oblique or 4-chamber views. Measurements of the VSD location and left and right ventricular orifices were taken. The selected device was typically 1–2 mm larger than the smallest part of the defect. In the retrograde approach, the defect was crossed from the LV using a J-angled tip guide wire, with a Judkin Right or cut pigtail catheter to establish access. The delivery sheath was advanced over the wire to the RV. The device was loaded onto the delivery cable and advanced to the sheath's tip under fluoroscopy, with the right disk deployed first, followed by the device's waist and left disk against the septum. The antegrade approach was used for small patients with large defects requiring a larger sheath or in cases of deficient aortic rims, insufficient distance to the aortic valve, trivial aortic valve regurgitation, or mild aortic valve prolapse. This involved an arterio-venous loop and a kissing technique to advance the delivery sheath through the femoral vein, across the defect, and into the LV or descending aorta. Left ventriculography and TTE were repeated before and after device release to evaluate position, closure, and valve competency. The device was released upon complete closure, with only a non-significant residual shunt and no new onset of aortic or tricuspid regurgitation or obstruction.

### Post-operative care and follow-up protocol

Patients were prescribed oral aspirin (3–5 mg/kg) once daily for 6 months. Endocarditis prophylaxis was administered as needed during the first 6 months, with continued antibiotic prophylaxis if incomplete closure was detected on follow-up. Before discharge, patients underwent a clinical examination, chest x-ray, 12-lead ECG, and echocardiogram. Uncomplicated cases were typically discharged 48 h post-procedure. Follow-up assessments, including clinical evaluations, ECGs, and TTE, were scheduled 1, 3, and 6 months after the procedure.

### Study outcomes

The procedure was considered successful if the device was properly implanted in an appropriate position without any major complications or a hemodynamically significant residual shunt. The residual shunt was defined as the presence of color flow around the device.

We classified residual shunts as significant or non-significant. A residual shunt was considered significant if it exceeded 30% of the patient's aortic diameter or was associated with a Qp/Qs ratio greater than 1.5 by hemodynamic catheterization (25, 26).

Complications were categorized as major or minor. Major complications included mortality, potentially fatal adverse events, or those requiring intervention (e.g., embolization, myocardial perforation, vascular rupture, severe residual shunt, severe hemolysis, aortic or tricuspid valvular damage, persistent atrioventricular block). Minor complications were typically resolved with medical management, such as issues with vascular access, mild hemolysis treated medically, transient atrioventricular block, or other conduction abnormalities not requiring pacemaker implantation, fever, or neurapraxia.

### Statistical analysis

Categorical variables were reported as frequency and percentage and continuous variables were represented as mean with standard deviations. The normality of measurements was assessed using the Shapiro–Wilk test. Statistical analyses for continuous variables were conducted using T-Test.

## Results

### Patients

A total of 151 patients underwent percutaneous VSD closure with the KONAR-MFO. The mean age of the patients was 55.4 ± 51.6 years (range, 6 months to 31 years), and the mean weight was 17.6 ± 11.9 kg (range, 5–86). Persistent clinical symptoms of congestive heart failure including failure to thrive, recurrent chest infection, difficulty feeding, and easy fatigability were present at the time of assessment despite anti-failure treatment in 32 patients (21.2%) (Table 1).

**Table 1:** Baseline patient demographics and clinical characteristics.

Baseline demographics	Overall, *n* = 151		Perimembranous VSD, *n* = 94		Muscular VSD, *n* = 57	
Age (months), mean (±SD)	55.4 ± 48.4		53.2 ± 54.1		58.7 ± 38.3	
Male gender, *N* (%)	72	(47.7)	41	27.2	31	20.5
Weight (kg) (*N* (%)	17.6 ± 11.9		17.1 ± 15.4		18.4 ± 10.9	
≤5 kg	5	(3.31)	3	(2.0)	2	(1.3)
6–10 kg	40	(26.5)	24	(15.9)	16	(10.6)
>10 kg	106	(70.2)	67	(44.4)	39	(25.8)
Height (cm)	102.6 ± 25.6		101.9 ± 26.9		104 ± 25.2	
Body mass index (kg/m^2^), mean (±SD)	15.2 ± 3.1		11.6 ± 3.8		20.6 ± 3.5	
Pauci-/asymptomatic, *N* (%)	119	(78.9)	75	(49.7)	44	(29.1)
Indication of intervention:						
Clinical symptoms, *N* (%)	32	(21.2)	18	(11.9)	14	(9.3)
LVEDD *z*-score ≥2.0, or left atrium-to-aortic diameter ratio >1.5, *N* (%)	115	76.6	72	47.7	43	28.5
Aortic valve prolapse, *N* (%)	3	(2.0)	3	(2.0)	0	(0.0)
History of infective endocarditis, *N* (%)	1	(0.7)	1	(0.7)	0	(0.0)
LVEDD *Z*-score, mean (±SD)	1.8 ± 3.5		1.7 ± 3.6		1.9 ± 2.5	
Defect left ventricular diameter (mm), mean (±SD)	8.9 ± 2.4		8.7 ± 2.6		9.2 ± 1.9	
Defect right ventricular diameter (mm), mean (±SD)	4.8 ± 1.5		4.7 ± 1.5		4.9 ± 1.4	
Echocardiographic systolic PAP (mmHg), mean (±SD)	32.8 ± 6.2		32.6 ± 6.5		33.1 ± 5.9	
Echocardiographic mean PAP (mmHg), mean (±SD)	18.9 ± 3.1		18.8 ± 3.1		19.1 ± 3.0	
Indirect gerbode shunt, *N* (%)	5	(3.3)	2	(1.3)	3	(2.0)
Mild RVOTO, *N* (%)	2	(1.3)	0	(0.0)	2	(1.3)
Other associated lesions, *N* (%)	16	(10.6)	8	(5.3)	8	(5.3)
Required concomitant interventions, *N* (%)	11	(7.3)	7	(4.6)	4	(2.6)
ASD	4	(2.6)	3	(2.0)	1	(0.7)
PDA	5	(3.3)	2	(1.3)	3	(2.0)
LPA stenosis	1	(0.7)	1	(0.7)	0	(0.0)
Pericardial effusion	1	(0.7)	1	(0.7)	0	(0.0)
Not requiring concomitant interventions, *N* (%)	5	(3.3)	1	(0.7)	4	(2.6)
Small ASD	2	(1.3)	0	(0.0)	2	(1.3)
Tiny PDA	3	(2.0)	1	(0.7)	2	(1.3)

LV, left ventricle; LA, left atrium; LVEDD, left ventricular end-diastolic diameter; PAP, pulmonary artery pressure; RVOTO, Right ventricular outflow tract obstruction; ASD, atrial septal defect; PDA, patent ductus arteriosus; LPA, left pulmonary artery.

### Defects

The pre-procedural assessment was performed by TTE in all patients but only 14 patients required further assessment by TEE. Concomitant defects that needed interventions in the same setting were present in 11 (7.3%) patients (Table 1). No extra-cardiac comorbidities were found.

### Procedure

**Figure 2:**
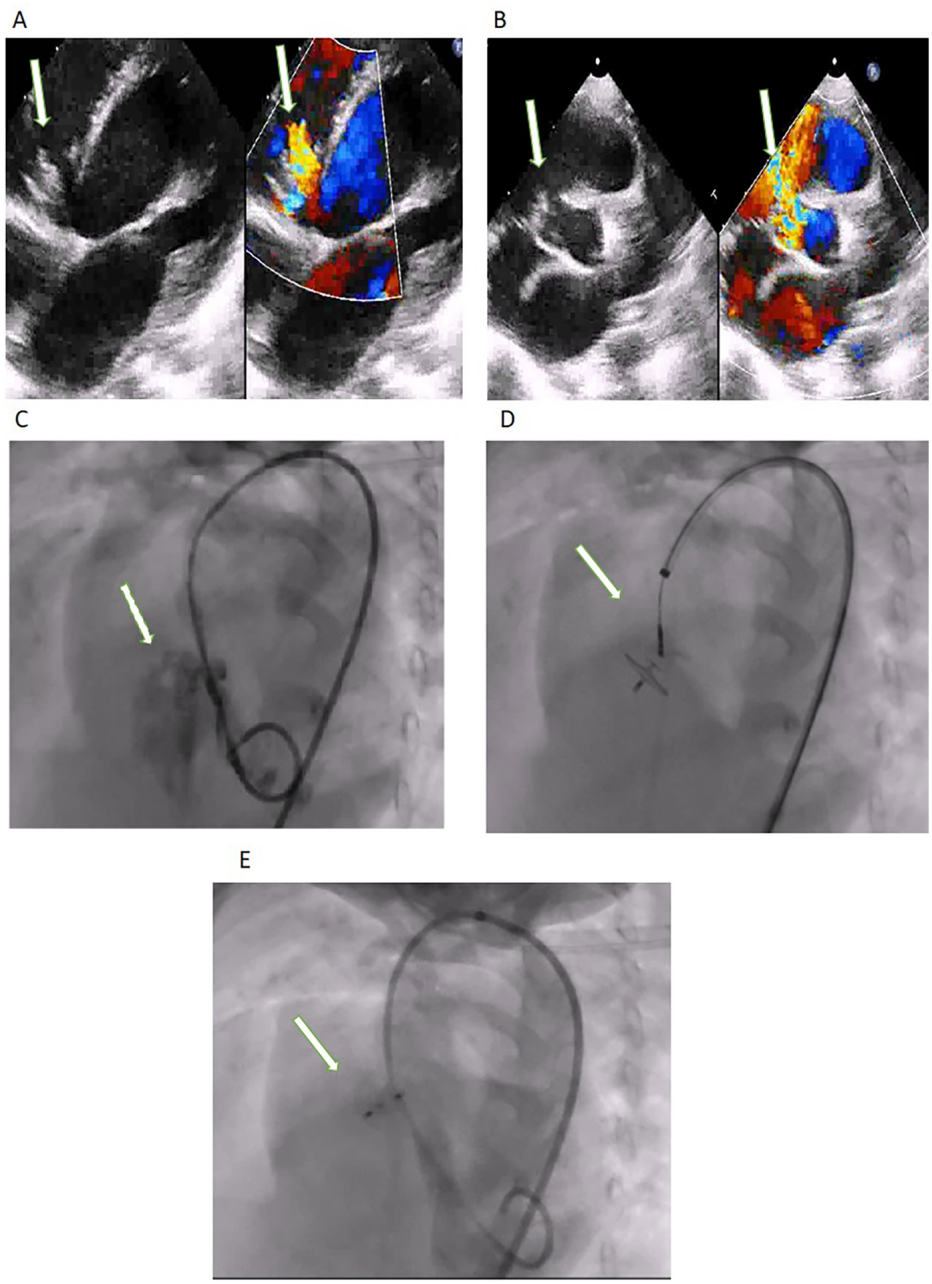
**(A)** TTE modified apical four-chamber with Doppler color and **(B)** parasternal short axis view with Doppler color showing the perimembranous VSD (arrow). **(C)** Left Ventricular Angiography: the arrow shows the VSD. **(D)** Retrograde approach showing device occluding the defect with the delivery cable attached to the LV disc. Post-device closure **(E)** left ventricular angiogram showing good device position (arrow) and no significant residual flow.

**Figure 3:**
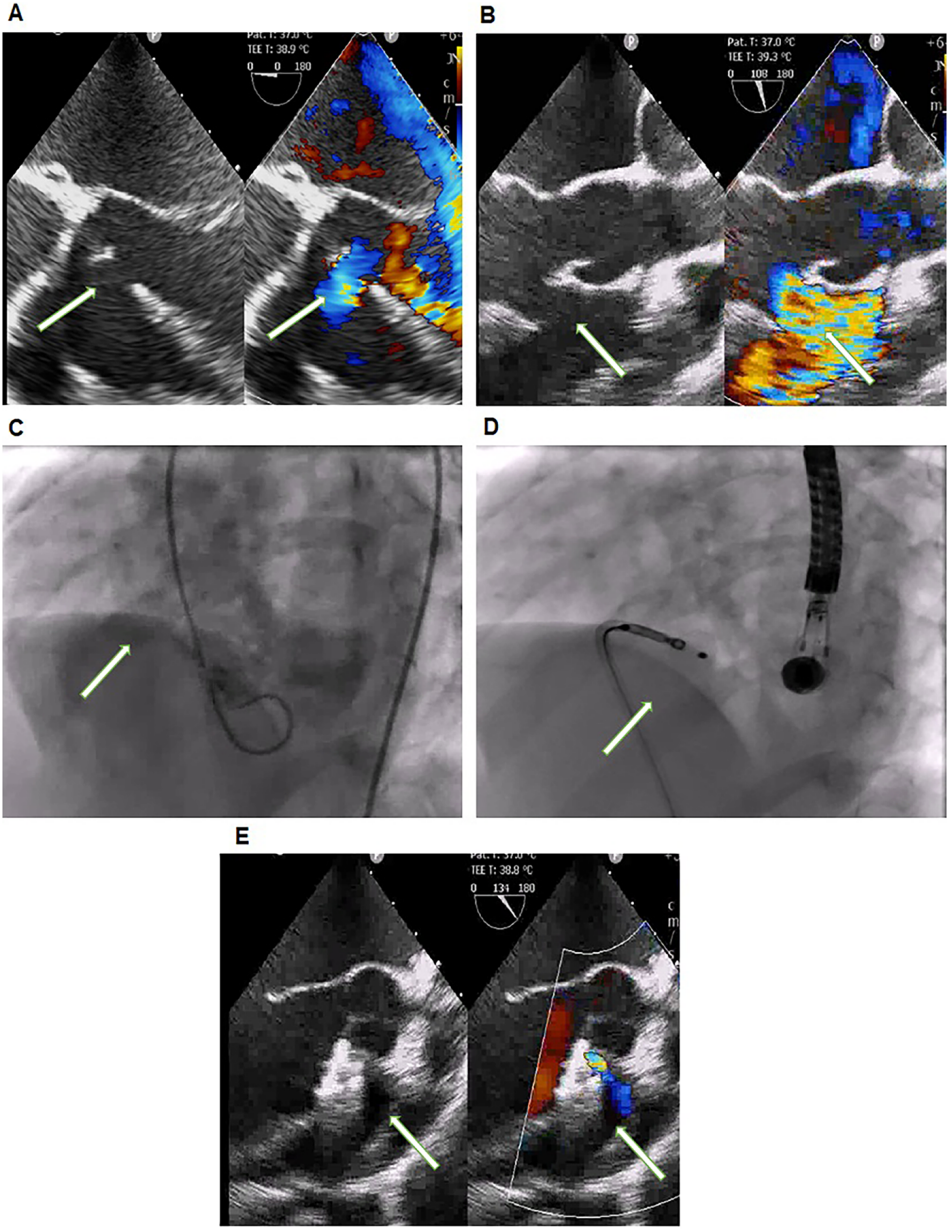
**(A)** TTE 0 degrees view with Doppler color and **(B)** TEE 110 degrees with Doppler color showing the perimembranous VSD (arrow). **(C)** Left Ventricular Angiography: the arrow shows the VSD. **(D)** The antegrade approach shows the device occluding the defect with the delivery cable attached to the RV disc. Post-device closure in **(E)** TEE 135 degrees with Doppler color showing a stable device with no significant residual flow (arrow).

The antegrade route was used in 17 (11.3%) patients (Figure 2) of which only 9 (6%) needed complete arterio-venous loop formation. The failed aborted procedure followed an antegrade approach. The cases that underwent concomitant procedures were excluded from the calculations of procedure duration, radiation time, radiation dose, and contrast dose (Table 2). Compared to the antegrade approach the retrograde approach (Figure 3) resulted in a shorter procedure duration (40.2 ± 15.5 min vs. 72.30 ± 39.1 min) (*p* = 0.01), fluoroscopy time (11.8 ± 6.1 min vs. 20.97 ± 12.9 min) (*p* = 0.03), smaller radiation dose (0.2 ± 0.2 Gy vs. 0.41 ± 0.4 Gy) (*p* = 0.07) and contrast dose (31.1 ± 20.8 ml/kg vs. 33.5 ± 26.2 ml/Kg) (*p* = 0.28).

**Table 2:** Procedural details.

Procedural details	Overall, *n* = 151		Perimembranous VSD, *n* = 94		Muscular VSD, *n* = 57	
Selected route						
Antegrade, *N* (%)	17	(11.3)	11	(7.3)	6	(4.0)
Retrograde, *N* (%)	134	(88.7)	83	(55.0)	51	(33.8)
Delivery sheath						
5 FR, *N* (%)	91	(60.3)	52	(34.4)	39	(25.8)
6 FR, *N* (%)	49	(32.5)	36	(23.8)	13	(8.6)
7 FR, *N* (%)	11	(7.3)	6	(4.0)	5	(3.3)
Occluder size						
6 × 4, *N* (%)	18	(11.9)	11	(7.3)	7	(4.6)
7 × 5, *N* (%)	35	(23.2)	21	(13.9)	14	(9.3)
8 × 6, *N* (%)	38	(25.2)	28	(18.5)	10	(6.6)
9 × 7, *N* (%)	28	(18.5)	18	(11.9)	10	(6.6)
10 × 8, *N* (%)	24	(15.9)	12	(7.9)	12	(7.9)
12 × 10, *N* (%)	8	(5.3)	4	(2.6)	4	(2.6)
Procedure duration (min) mean (±SD)	44.0 ± 22.0		51.8 ± 30.4		44.8 ± 21.3	
Radiation time (min) mean (±SD)	13.1 ± 9.0		13.0 ± 9.6		13.5 ± 8.0	
Radiation dose (Gy) mean (±SD)	0.21 ± 0.23		0.2 ± 0.2		0.4 ± 0.8	
Contrast dose (ml/kg) mean (±SD)	31.2 ± 21.4		28.8 ± 17.9		33.2 ± 22.3	

### Outcomes and follow-up

During our procedures, we encountered one major complication requiring surgical retrieval and VSD closure due to device dislodgment. This incident involved a 10-month-old infant with a body weight of 6.5 kg and a large PM VSD measuring 12 mm on the left ventricular (LV) side, accompanied by a thin aneurysm on the right ventricular (RV) side with residual shunting of 8 mm diameter. The selected 12 × 10 device was loaded but proved unstable, leading to dislodgement and embolization to the RV where it was retrieved. Given the patient's weight, a larger device was not viable. There were no other events of device migration or need for surgery, nor any fatal adverse events.

The follow-up ranged from 6 to 35 months (11 ± 9.7 months) with 94 patients having 6 or more months of follow-up.

One patient (0.7%) developed a significant residual shunt that required placement of an additional device in a subsequent session, with no complications. This 10-month-old infant of 6 Kg initially presented with a large non-restrictive PMVSD with an RV end of about 5 mm in diameter and intractable heart failure symptoms. An 8/6 device was deployed but follow-up showed a significant residual shunt. However, despite the significant residual shunting, the patient's condition improved over time and he gained weight.

The non-significant residual shunts (*n* = 32, 21.2%) were small, showed no significant volume load or incidence of hemolysis over time and the device stayed in place (Table 3).

**Table 3:** Outcomes and follow-up.

Outcomes and follow-up	Overall, *n* = 151		Perimembranous VSD, *n* = 94		Muscular VSD, *n* = 57	
Success rate	149	(98.7)	92	(60.9)	57	(37.7)
Hospital stay (Days), mean (±SD)	1.2 ± 0.6		1.2 ± 0.7		1.2 ± 0.6	
Device embolization	1	(0.7)	1	(0.7)	0	(0.0)
Residual shunt	33	(21.9)	20	(13.2)	13	(8.6)
Significant	1	(0.7)	1	(0.7)	0	(0.0)
Non-significant	32	(21.2)	19	(12.6)	13	(8.6)
Newly acquired valve regurgitation	16	(10.6)	12	(7.9)	4	(2.7)
AR (trivial-mild)	11	(7.3)	9	(6.0)	2	(1.3)
TR (Moderate-severe)	5	(3.3)	3	(2.0)	2	(1.3)
Adverse EP events	5	(3.3)	3	(2.0)	2	(1.3)
Nodal rhythm	3	(2.0)	2	(1.3)	1	(0.7)
Intermittent heart block	1	(0.7)	0	(0.0)	1	(0.7)
Severe bradycardia and asystole	1	(0.7)	1	(0.7)	0	(0.0)
Total vascular complications	13	(8.6)	6	(4.0)	7	(4.6)
Local Hematoma	1	(0.7)	0	(0.0)	1	(0.7)
Peripheral ischemia	12	(7.9)	6	(4.0)	6	(4.0)
Responding to anticoagulation	2	(1.3)	1	(0.7)	1	(0.7)
Responding to Thrombolytics	9	(6.0)	5	(3.3)	4	(2.6)
Not responding to medical treatment	1	(0.7)	0	(0.0)	1	(0.7)

AR, Aortic regurgitation; TR, Tricuspid regurgitation.

Three patients experienced transient events during the procedure in the form of heart block (*n* = 1, 0.7%) and nodal rhythm (*n* = 2, 1.3%), which resolved spontaneously (Table 3). One patient developed a nodal rhythm that persisted for 48 h after the procedure and resolved after steroid therapy, with no need for temporary or permanent pacemaker insertion.

Severe sinus bradycardia developed in one patient (0.7%) which progressed to asystole during the procedure due to atrial overstretch while completing the arteriovenous loop. This 6 kg infant required CPR for 30 s followed by complete recovery and no further adverse events.

All vascular complications occurred in the retrograde approach. Only one patient didn't respond to both anticoagulation and thrombolytic therapy and developed chronic occlusion for which he was discharged on subcutaneous heparin. Follow-up duplex ultrasound after 6 months showed total recanalization of the affected limb.

## Discussion

### Success rate

Our results show an overall success rate of 98.7%, and excellent short- and mid-term follow-up, reaffirming the excellent MFO results (9, 19, 27), even among selected infants with low body weight, and those with large defects.

Our patient cohort consisted of a fairly young population, similar to previous studies, in which 29.8% were below 10 kg (10). We encountered embolization of one device and one significant residual shunt. Device embolization occurred in a young child with low weight presenting with a large PM VSD with a thin aneurysm due to the downsizing of the device to accommodate the patient's low weight. The retrieval of the Konar-MFO after embolization was easy, due to the device's high flexibility and softness, as well as the smaller delivery sheath and the presence of a removable screw on both device discs.

### Residual shunt

Only one patient developed a significant residual shunt, with non-significant shunts in 32 patients (21.2%), similar to previous studies (19, 27, 28).

The occurrence of a significant residual shunt in a small child in which the weight constrained the use of a larger device. Immediate smoky small intra-device residual shunts were common, especially in the MFO device sizes that do not contain a PTFA membrane enhancing the process of clotting, but closed spontaneously within the first 24 h after the procedure with no hemodynamic significance.

With a high success rate of occlusion and a low complication incidence, the MFO offers a secure and efficient alternative to surgery (10, 20, 27, 29) and other occlusion devices (19, 20, 27–30), even in the smallest patients (10, 20, 27, 29). However, careful patient and device selection is vital to minimize complications in VSD closure, especially in young children. Comprehensive long-term data from larger prospective studies are essential to affirm the device's efficacy and safety.

### Valve injuries

In this cohort, newly acquired AVR only occurred in 7.3% of patients and remained trivial to mild in similarity to previous studies (9, 18). However, severe injury to the aortic valve might occur with the MFO in suboptimal anatomies such as aortic valve prolapse associated with absent aortic rim and absent of tapered aneurysm (31).

Newly acquired TV regurgitation was less common than AVR (3.3%) but more severe. Transthoracic echocardiography (TTE) and, in complex cases, transesophageal echocardiography (TEE) assessments of both tricuspid and aortic valves are imperative to identify and assess valvular regurgitation mechanisms before and after device deployment.

In our series, the positioning of the right disc was continuously evaluated by echocardiography before deployment. Any signs of tricuspid valve interference prompted immediate sheath retraction and repositioning of the right disc close to the septum before final deployment. This strategic readjustment notably reduced the incidence of significant iatrogenic tricuspid regurgitation post-procedure.

### Minor complications

In our series, the adverse electrophysiological events were transient and resolved spontaneously, with only one patient requiring steroid therapy. The MFO is known for its lower rate of arrhythmias (9, 15, 21) and heart block (17) compared to other devices, due to the soft structure and flexible design which minimizes trauma to the conduction tissue and lowers radial stress force, allowing significant oversizing of the device without injury to any adjacent structures (19, 32).

All vascular complications observed were associated with the retrograde approach, in which arterial access necessitated an enlargement in size to accommodate the delivery sheath, leading to possible vascular complications.

### The route

In our study, a retrograde approach was successful in the majority of patients (88.7%). The versatility of the MFO, adaptable to both antegrade and retrograde vascular approaches, expands its usability. However, the retrograde approach is preferred when feasible, reserving the antegrade method for challenging complex cases, particularly in small children or defects with limited aortic rim to prevent aortic valve injury.

Avoiding the antegrade approach especially if arteriovenous looping is needed can enable an easier and faster procedure, enhancing efficiency, reducing risks, safeguarding the aortic valve, minimizing radiation, and shortening general anaesthesia time.

#### Suggested future modifications and possible potentials

Based on our experience with the device, we propose modifications to enhance its efficacy and broaden its utility across different defect types. We recommend a slight reduction in the size of the right ventricular disk compared to the left ventricular disk to minimize tricuspid valve and conduction system interference. Additionally, larger device designs with left ventricular disk diameters up to 20 mm could effectively address bigger defects. To tackle challenges in adult patients, we suggest longer delivery sheaths suitable for retrograde procedures.

Despite limited experience with doubly committed sub-arterial VSDs due to their rarity in our region (33, 34) we anticipate difficulties due to the acute angle between the selected occluder and the delivery system, hindering precise device positioning as well as the risk of aortic valve prolapse-related complications. The MFO's flexible composition and versatile deployment options could offer improved maneuverability and reduced risks of aortic valve interference.

#### Study limitations

This study included a limited number of participants spanning a wide age and weight range. In addition, the follow-up duration was relatively short, which is critical for assessing long-term complications. The lack of extended follow-up may hinder our ability to fully understand the long-term outcomes and potential risks associated with the procedure.

## Conclusion

Percutaneous closure of VSDs using the Lifetech™ Konar-MF device is feasible, safe, and effective, through the antegrade as well as the retrograde approach, with excellent results at short and mid-term follow-up, even in large defects in small pediatric patients. Larger studies with long-term data are needed to guide future recommendations.

## Data Availability

The raw data supporting the conclusions of this article will be made available by the authors, without undue reservation.
